# European guidelines on quality criteria for diagnostic radiographic images of the lumbar spine – an intra- and inter-observer reproducibility study

**DOI:** 10.1186/s12998-019-0241-3

**Published:** 2019-05-01

**Authors:** Klaus Doktor, Maria Lind Vilholm, Aldis Hardardóttir, Henrik Wulff Christensen, Jens Lauritsen

**Affiliations:** 1 Research Unit of Clinical Biomechanics, University of Southern Denmark, Campusvej 55, 5250 Odense M, Denmark; 2 Private chiropractic practice, Back Center Midwestern Jutland, Dalgas Allé 2A, 7400 Herning, Denmark; 3 Private chiropractic practice, Reykjavik, Iceland; 4 Nordic Institute of Chiropractic and Clinical Biomechanics, Campusvej 55, 5230 Odense M, Denmark; 5 Institute of Clinical Medicine, University of Southern Denmark, Odense, Denmark; 6 Orthopedic Department, Odense University Hospital, Odense, Denmark

**Keywords:** Agreement, Reliability, Reproducibility, EU-quality criteria, EU-guidelines, Lumbar spine, Radiographs, X-rays, Radiography, Chiropractor, Imaging, Primary practice, Primary care

## Abstract

**Background:**

The Commission of the European Communities has published guidelines to be used as a gold standard for quality assessment of diagnostic radiographic images. Image quality and radiation dose must be monitored and optimally balanced for diagnostic purposes on patients. The objective of the current study was to assess intra- and inter-observer reproducibility in less experienced observers using the proposed European Guidelines on Quality Criteria for Diagnostic Radiographic Images in a quality assessment of lumbar spine radiographs in primary chiropractic practice in Denmark.

**Methods:**

Two observers initially evaluated lumbar spine radiographs randomly selected from fifty chiropractic clinics, all connected to the national PACS server (KirPACS) in Denmark. All evaluations were performed twice by both observers using a four-week interval and for compliance with the European Quality Criteria for Diagnostic Radiographic Images. Inter- and intra-observer reproducibility was calculated using kappa statistics. In the interpretation of the kappa coefficient, the standards for strength of agreement reported by Landis and Koch were followed.

**Results:**

The strength of the inter-observer agreement of general image quality at baseline ranged from moderate agreement (*k* = 0.47) to substantial agreement (*k* = 0.68). After four weeks, the inter-observer agreement still ranged from moderate agreement (*k* = 0.59) to substantial agreement (*k* = 0.71), but with increased agreement for both kappa coefficients. In relation to intra-observer agreement of general image quality, the strength for observer A ranged from moderate (*k* = 0.58) to substantial (*k* = 0.72) and the strength for observer B overall was substantial (*k* = 0.63–0.75).

**Conclusion:**

The European Guidelines on Quality Criteria for Diagnostic Radiographic Images are considered a gold-standard and used in a method for quality assurance within the Danish chiropractic profession. The inter-rater and intra-rater agreements in this study, using the CEC-criteria, were found mostly acceptable. With appropriate attention to clear understanding of the individual criteria and sufficient training, this method is found to be reliable, even using less experienced observers, to carry out Diagnostic Radiographic Image Quality-assurance in primary care settings.

**Electronic supplementary material:**

The online version of this article (10.1186/s12998-019-0241-3) contains supplementary material, which is available to authorized users.

## Background

In Denmark, primary chiropractic practices consist of approximately 249 individual clinics [1], the vast majority of which have their own radiographic imaging systems; there are approximately 170 clinics using digitalized radiographic systems, mostly Computed Radiography and to less extent Direct Radiography [2].

Historically, chiropractors in Denmark have had the rights and privileges to operate their own x-ray units. In 2008 a national Picture Archiving and Communication System (PACS) was established at the Nordic Institute for Chiropractic and Clinical Biomechanics (NIKKB), University of Southern Denmark. The system (KirPACS) was initially a standard PACS-system but has since been developed and expanded with functionalities to include documentation for various quality control and quality assurance activities including a diagnostic second opinion service for participating clinics. This unique system is an example of a cost-efficient concept to monitor radiation exposure doses and image quality control procedures.

A protocol for quality assessment of lumbar spine radiographs is proposed in the publication by the Commission of the European Communities: EUR Report 16,260 “European Guidelines on Quality Criteria for Diagnostic Radiographic Images” [3]. These guidelines were used in a European-wide trial on the use of quality criteria between the various professionals and authorities involved in diagnostic radiology [4]. The image criteria specify important anatomical structures that should be visible in a radiograph to aide accurate diagnosis. Some of these criteria depend fundamentally in correct positioning and cooperation of the patient, whereas others reflect technical performance of the imaging system. A qualitative guide to the necessary degree of visibility of these essential structures is provided. They are a gold standard for quality assessment of radiographic images and must be used in any type of measures of image quality and dose relations (see Additional file 1: A and B). In the original report the levels of agreement among observers are not clearly documented. This is also the case for other similar studies [5].

Among Danish chiropractors, the European Guidelines are used in an ongoing quality assurance program to be performed once every 2 years. Studies of the reproducibility of the image quality criteria are very limited in numbers in the literature and we found it relevant to report on our findings, since this is a crucial element in optimizing the diagnostic gain for patients.

## Objectives

The objective of this study was to assess the inter- and intra-observer reproducibility in less experienced observers using an evaluation protocol conforming to the “European Guidelines on Quality Criteria for Diagnostic Radiographic Images” proposed by the European Commission Study Group.

## Methods

### Design

The present study is an intra- and inter-observer reproducibility study of the CEC-guidelines using repeated measurements of individual lumbar spine radiographs.

### Study population

To establish the level of intra- and inter-observer reproducibility, data were extracted from readings of fifty lumbar spine radiographs. The study materials were randomly selected, anonymized and numbered from fifty chiropractic clinics, retrieved from an archive of approximately 29,400 lumbar spine studies produced by Danish chiropractors in 2015–2016 [2]. The focus was purely on quality of images and was part of a quality assurance program, as required biannually by Danish law [6]. The study materials were blinded to the observers with respect to any personal information and no diagnostic information was recorded.

### Instruments

For the image analysis, the observers were using a digitized format of the CEC Quality Criteria and all images were retrieved from KirPACS using the image viewer Osirix v. 5.7.1 for Mac [7]. The results were tabulated directly into forms made in the software program Epidata v.2.0.7.22 r547 [8].

Diagnostic monitors (2 million pixels) from Barco (MDNC 2121 color led display) [9] were used for the entire image evaluation process. Monitors had passed acceptance tests according to Danish regulations.

### Observers and training

The two observers were both licensed chiropractors by the Danish National Board of Health and were in their first 2 years of clinical practice. They were purposely selected with limited clinical experience in accordance with the study objective, but both had a high interest in radiology in general. The observers received adequate introduction in the use of the CEC quality criteria. Initially ten lumbar spine series were evaluated in a joint session to ensure consensus in understanding the criteria and the evaluation process. An experienced supervisor attended this initial session to guide consensus. These studies were excluded from use in the reproducibility study.

### Blinding

The observers were blinded to any personal patient information such as: Name, birth date, social security number, image accession number, report of findings and clinic identification. They worked independently and had no access to any previous readings or images. Every effort was done to exclude any confounding factors that could compromise our observers and the results. The observers were given 2 weeks to finish their evaluations and could log on and off to access the images any time they wished.

### General image quality assessment

After consensus was reached, fifty lumbar spine series were evaluated by blinded and independent observers. After more than four weeks, trying to minimize any recognition of image features, both observers re-evaluated the same fifty image studies for the intra-observer reliability evaluation. Observers could pick images in any order, consecutive or random. The images were not re-randomized for the second evaluation.

The general image quality assessment followed the scoring principles described in the additional file 1. To determine the level of reproducibility between the two observers, we used the General Image Acceptability-scores for each of the three standard lumbar projections providing three variables from each observer for each radiographic series read and scored (See Additional file 1: A): 1.3.1 Acceptability Lumbar AP/PA-projection, 2.3.1. Acceptability Lateral L1 to L4-projection and 3.3.1. Acceptability Lateral L/S-projection. For description of the scoring principles (see Additional file 1: B). General acceptability was scored based on impressions of overall noise, contrast, sharpness, collimation and patient positioning. Image quality/acceptability were initially scored using a scale from 0 to 3 points (0 = unacceptable; 1 = only acceptable under certain clinical conditions; 2 = probably acceptable, 3 = fully acceptable).

It is important to establish an acceptable level of agreement for the proposed method used to evaluate diagnostic image quality. Any procedure used in evaluations of performance must be validated to ensure reliable results. We therefore tested the intra- and inter-observer reproducibility of a general assessment of image quality using Kappa-statistics.

### Statistical analysis

For kappa statistics score-groups 0 and 1 were merged into a “not accepted group” and score-groups 2 or 3 were merged into an “accepted group”. All accepted images received 1 point and not accepted images received 0 point. This allowed us to calculate the intra- and inter-observer reproducibility by means of ordinary Kappa for binomial variables. Inter-observer reproducibility was analyzed using results from the first (baseline) evaluations. The ratings from each observer were cross-tabulated in Epidata Entry Client and agreement was measured using Cohens Kappa statistics in Stata. Results were expressed as Kappa values with standard errors and Z-scores indicated.

A Kappa value of 1 represents perfect agreement between the observers; whereas a value of 0 means that the results were obtained by chance. The Kappa values were interpreted according to the recommendations of Landis and Koch [10]. Values below 0.00 indicate poor agreement; 0.00–0.20 slight agreement; 0.21–0.40 fair agreement; 0.41–0.60 moderate agreement; 0.61–0.80 substantial agreement and a Kappa above 0.81 indicated almost perfect agreement. Kappa values over 0.6 are considered reliable.

Statistical analysis was performed using the STATA 14 for Windows, Stata Corporation, USA [11]; Microsoft Excel 2010, Microsoft Office Package, Microsoft Corporation, USA [12]; Epidata Entry Client and Epidata Manager [8].

## Results

A total of fifty lumbar spine radiographs were evaluated at baseline by two independent observers. After 4–6 weeks, the radiographs were re-evaluated by both observers to determine the level of intra-observer reproducibility.

### Inter-observer reproducibility

In Table 1, percent agreement, expected agreement and Kappa values are presented at baseline.

**Table 1:** Inter-observer agreement for general image quality assessment of the lumbar spine (baseline) *n* = 50

Lumbar projection	Agreement	Expected Agreement	Kappa (*k*)	Standard Error	Z	Probability>Z
AP/PA	82.00%	62.48%	0.5203	0.1412	3.68	0.0001
Lat. L1-L4	84.00%	49.60%	0.6825	0.1374	4.97	0.0000
Lat. L5-S1	78.00%	58.28%	0.4727	0.1288	3.67	0.0001

In Table 2, percent agreement, expected agreement and Kappa values are presented > 4 weeks.

**Table 2:** Inter-observer agreement for general image quality assessment of the lumbar spine (> 4 wks) *n* = 50

Lumbar projection	Agreement	Expected Agreement	Kappa (*k*)	Standard Error	Z	Probability >Z
AP/PA	86.00%	66.00%	0.5882	0.1303	4.51	0.0000
Lat. L1-L4	86.00%	51.64%	0.7105	0.1370	5.19	0.0000
Lat. L5-S1	88.00%	60.76%	0.6942	0.1338	5.19	0.0000

### Intra-observer reproducibility

In Table 3, percent agreement, expected agreement and Kappa values for intra-observer agreements are presented for observer’s A and B.

**Table 3:** Intra-observer agreement for image quality evaluations of the lumbar spine

	Agreement	Expected Agreement	Kappa (*k*)	Standard Error	Z	Probability >Z
Observer A						
AP/PA	86.00%	66.32%	0.5843	0.1350	4.33	0.0000
Lat. L1-L4	86.00%	49.52%	0.7227	0.1359	5.32	0.0000
Lat. L5-S1	90.00%	65.24%	0.7123	0.1352	5.27	0.0000
Observer B						
AP/PA	86.00%	62.00%	0.6316	0.1358	4.65	0.0000
Lat. L1-L4	88.00%	51.20%	0.7541	0.1370	5.50	0.0000
Lat. L5-S1	84.00%	55.72%	0.6387	0.1356	4.71	0.0000

### General image quality/acceptability

The strength of the inter-observer agreement at baseline ranged from moderate agreement (*k* = 0.47) to substantial agreement (*k* = 0.68). After four weeks, the observers read the images one more time and now the inter-observer agreement increased although still ranging from moderate agreement (*k* = 0.59) to substantial agreement (*k* = 0.71).

In relation to intra-observer agreement of image quality, the strength for observer A ranged from moderate (*k* = 0.58) to substantial (*k* = 0.72) and the strength for observer B overall was substantial (*k* = 0.63–0.75).

Kappa values > 0.6 is accepted as reliable.

## Discussion

In this study of intra- and inter-observer reproducibility when using the CEC-criteria to evaluate radiographic image quality, we also wanted to find out if clinicians in primary chiropractic practice can be expected to reach acceptable levels of agreement, when used as observers. Validating the use of the CEC-guidelines among clinicians with various degrees of experience is important, since this method is a key element in the optimization of image quality among chiropractors throughout Denmark and because resources are allocated to continued post-graduate education in image quality assurance. Keeping in mind our objective, we intentionally left observers with a brief introduction, instead of extensive training in the use of image quality criteria in an attempt to mimic a realistic clinical setting. We observed that after the first set of fifty evaluations our observers showed mildly improved levels of reliability. According to the methods recommended by Landis and Koch, our results can be rated with moderate to substantial agreement.

### Intra-observer reproducibility

Except for a borderline Kappa value for observer A for the AP/PA lumbar projection, all other scores were considered reliable. Since the two lateral projections are traditionally combined into one image extending from levels Th12-S2, in most chiropractic clinics, it is not surprising that these two evaluations have almost identical Kappa scores. It is in full accordance with the CEC-document that a standard lumbar spine series can consist of only two, instead of three projections, although this may require the use of a compression belt to ensure even density.

The observers of this study were in their first years of practice and had never worked with image quality assessments, nevertheless it was possible to accomplish mostly acceptable agreement. We would expect experienced observers to achieve a higher level of agreement with this evaluation system, as seen before in the publication by Maccia et al. [4].

### Inter-observer reproducibility

If we exclude the first baseline Kappa values for inter-observer reliability and use Kappa-values found at four weeks, our results indicate that primary care clinicians should be able to reliably apply this system with sufficient initial training. A reason for higher levels of disagreement in the first quality assessments was probably due to variations in perception of the image quality criteria. The lateral lumbar spine projections achieved better agreement than the AP/PA projection due to better visibility of structures. The lumbar spine AP/PA projection is more difficult to interpret, due to many superimposed structures and the lumbar curve. Some criteria are very clearly defined whereas others lack sharp definitions leaving room for interpretation, e.g., “important image details” (Appendix B, 2.2) is sharply defined for the lateral L1-L4 lumbar projection as: “Visually details down to 0.5 mm. at 3^rd^ lumbar vertebral body, ventral edge”; whereas for the AP/PA lumbar projection the definition is less specific: “Visually details down to 0.3-0.5 mm”; and for the lateral L5/S1 lumbar projection the definition is: “Linear and reticular details down to 0.5 mm. in width”. It would likely strengthen the Kappa scores, if we tightened the criteria interpretations by clarifying definitions (especially the AP/PA projection) for future quality evaluations.

The repeated evaluations after four weeks increased the agreement overall, which was concluded as likely to be due to gained experience by the observers during the study. It is important to allow observers to practise the evaluations in an initial trial to reach consensus. Our results indicate that more training of observers prior to initiating the study could have improved overall reliability.

In a study by Inah et al. of pelvic radiography image quality in a Nigerian teaching hospital, image evaluations were based on the Commission of European Communities (CEC) criteria, and an average Kappa value, *k* = 0.60 (0.36–0.76) for inter-observer reproducibility between two radiologists was reported [13]. The study included evaluation of 7 CEC quality criteria of the pelvis, whereas in comparison, we used a general overall assessment of lumbar spine image quality in our study and found Kappa values ranging from, *k* = 0.47–0.68. In a previous report of lumbar spine radiographic image quality among Danish chiropractic clinics, we concluded that a general quality assessment, as described above, remained in good consistency with the results of evaluations of 22 specific lumbar spine quality criteria. We showed a correlation-coefficient, r = 0.72–0.83, in this earlier study, indicating a clear positive correlation (unpublished report from NIKKB in 2000 by K. Doktor, N. Grunnet-Nilsson and C. Lebouef-Yde). This further emphasizes the usefulness of the criteria in a practical clinic setting.

In another study of image quality Tesselaar et al. compared lumbar spine radiographs in two different settings: Sensitivity class 400 (less noise) and sensitivity class 560 (more noise). They concluded that higher image quality produced higher inter-observer reliability, AC1 = 0.72 vs. 0.57 [14]. This is relevant to point out, since we, for economic reasons, used an open source image viewer for all image assessments.

There is limited data regarding the impact experience has on the reliable assessment of image quality standards. However, if we consider that experience in assessing diagnostic images for quality offers some similarities to assessing them for pathology (when using preset diagnostic search criteria), then studies looking at the effect of experience on reliability in diagnostic assessment may give us insight into its effect on reliable image quality assessment. Assendelft et al. found acceptable reproducibility among Dutch chiropractors evaluating primarily unspecific radiographic image findings. Intra-observer reliability was higher than inter-observer reliability [15]. Taylor et al. compared medical and chiropractic students, clinicians and radiology specialists and found higher reliability with more experienced observers evaluating radiographs. Specialists obtained the best results with no differences between the two professions [16]. Similar results were found by de Zoete et al. [17]. This suggests that greater experience in image assessment in general is likely to increase reliability across a variety of tasks.

## Limitations

Our study used an open source viewer. It is possible that if we had used a high-end image viewer our observers would have obtained a higher inter-observer agreement. Also, we didn’t re-randomize image studies for the second evaluation. This could possibly affect Kappa-scores in a favorable way.

## Conclusion and recommendations

The European Guidelines on Quality Criteria for Diagnostic Radiographic Images are considered a gold-standard and are used as a method for quality assurance within the Danish chiropractic profession. The inter-observer and intra-observer agreements in this study, using the CEC-criteria, were found to be mostly acceptable. With appropriate attention to a clear understanding of the individual criteria and sufficient training this method is found to be reliable, even using less experienced observers, to carry out Diagnostic Radiographic Image Quality-assurance in primary care settings.

Our results indicate that primary care clinicians should be able to reliably apply this system.

The CEC-quality criteria can be recommended for use in any radiographic lumbar spine imaging setting. Our results indicate that less experienced observers likely would benefit from training in an initial trial of at least 50 imaging studies.

## Additional file

Additional file 1: Overview of all lumbar image criteria, important details, general assessment and examples. (PDF 652 kb)

## Abbreviations

AP: From anterior to posterior
CEC: Council of the European Commission
CR: Computed Radiography
DR: Direct Radiography
EU: European Union
KirPACS: Danish Chiropractic Picture Archiving and Communication System
L/S: Lumbo-sacral junction
L1: First lumbar vertebra
L4: Fourth lumbar vertebra
L5: Fifth lumbar vertebra
NIKKB: Nordic Institute for Chiropractic and Clinical Biomechanics
PA: From posterior to anterior
PACS: Picture Archiving and Communication System
S1: First sacral vertebra

## References

[1] Danish Chiropractor Association. KiroFAKTA 2016.

[2] Nordic Institute of Chiropractic and Clinical Biomechanics. 2016.

[3] Carmichael JHE, Maccia C, Moores BM, Oestmann JW, Schbilla H, et al. European guidelines on quality criteria for diagnostic radiographic images: EU publication EUR 16260; 2000.

[4] Maccia C, Moores BM, Wall BF (1996). The 1991 CEC trial on quality criteria for diagnostic radiographic images: detailed results and findings.

[5] Gonzalez L, Vano E, Oliete S, Manrique J, Hernáez JM (1999). Report of an image quality and dose audit according to directive 97/43/Euratom at Spanish private radiodiagnostics facilities. Br J Radiol.

[6] Sundhedsstyrelsen. Bekendtgørelse om medicinske røntgenanlæg til undersøgelse af patienter, nr. 975 af 16. december 1998.

[7] Synaptivemedical. Clear Canvas. http://www.clearcanvas.ca.

[8] Lauritsen J, Bruus M. 2003-2018. Epidata. The Epidata Association, Odense, Denmark. http://www.epidata.dk.

[9] Barco. Nio 2MP (MDNC-2121) Specification sheet, Article number K9601651. http://www.Barco.com. Belgium.

[10] Landis JR, Koch GG (1977). An application of hierarchical kappa-type statistics in the assessment of majority agreement among multiple observers. Biometrics.

[11] Statacorp (2015). Stata statistical software: release 14.

[12] Microsoftcorp (2018). Microsoft Office 2010 package.

[13] Inah GB, Akintomide AO, Edim EE, Nzotta C, Egbe NO (2013). A study of pelvic radiography image quality in a Nigirian teaching hospital based on the Commission of European Communities (CEC) criteria. South Afr Radiogr.

[14] Tesselaar E, Dahlström N, Sandborg M (2016). Clinical audit of image quality in radiology using visual grading characteristics analysis. Radiat Prot Dosim.

[15] Assendelft WJ, Bouter LM, Knipschild PG, Wilmink JT (1997). Reliability of lumbar spine radiograph Reading by chiropractors. Spine.

[16] Taylor JAM, Clopton P, Bosch E, Miller K, Marcellis S (1995). Interpretation of abnormal Lumbosakral spine radiographs - a test comparing students, clinicians, radiology residents and radiologists in medicine and chiropractic. Spine.

[17] de Zoete A, Assendelft WJ, Algra PR, Oberman WR, Vanderschueren GM, Bezemer PD (2002). Reliability and validity of lumbosacral spine radiograph reading by chiropractors, chiropractic radiologists, and medical radiologists. Spine.

