# Supplementary material for: European guidelines on quality criteria for diagnostic radiographic images of the lumbar spine – an intra- and inter-observer reproducibility study
Source: Chiropr Man Therap. 2019 May 1;27:20. doi: 10.1186/s12998-019-0241-3 (PMC6492386; doi:10.1186/s12998-019-0241-3)
Supplement: Supplementary file 1 — Overview of all lumbar image criteria, important details, general assessment and examples. (PDF 652 kb) [file 12998_2019_241_MOESM1_ESM.pdf]

Additional files

A: Overview of all lumbar EU-image criteria, important details and general assessment

1. **LUMBAR SPINE AP/PA Projection (figure 1)**
   - **1.1. Image Quality Assessment Criteria**
     - 1.1.1. Visually sharp reproduction, as a single line, of the upper and lower-plate surfaces in the centered beam area.
     - 1.1.2. Visually sharp reproduction of the pedicles.
     - 1.1.3. Reproduction of the intervertebral joints.
     - 1.1.4. Reproduction of the spinous and transverse processes.
     - 1.1.5. Visually sharp reproduction of the cortex and trabecular structures.
     - 1.1.6. Reproduction of the adjacent soft tissues, particularly the psoas shadows.
     - 1.1.7. Reproduction of the sacro-iliac joints.
   - **1.2. Important Image Details**
     - 1.2.1. Visually details down to 0.3-0.5 mm.
   - **1.3. General Assessment**
     - 1.3.1. Image acceptability.

2. **LUMBAR SPINE Lateral L1-L4 Projection (figure 2)**
   - **2.1. Image Quality Assessment Criteria**
     - 2.1.1. Visually sharp reproduction of the upper and lower-plate surfaces, represented as lines with the resultant visualization of the intervertebral spaces.
     - 2.1.2. Full superimposition of the posterior vertebral edges.
     - 2.1.3. Reproduction of the pedicles and the intervertebral foramina.
     - 2.1.4. Visualization of the spinous processes.
     - 2.1.5. Visually sharp reproduction of the cortex and trabecular structures.
   - **2.2. Important Image Details**
     - 2.2.1. Visually details down to 0.5 mm. at 3rd lumbar vertebral body, ventral edge.
   - **2.3. General Assessment**
     - 2.3.1. Image acceptability.

3. **LUMBAR SPINE Lateral L5/S1 Projection (figure3)**
   - **3.1. Image Quality Assessment Criteria**
     - 3.1.1. Reproduction by tangential production of the inferior end plate of L5 and the superior end plate of S1.
     - 3.1.2. Visualization of the spinous process of L5.
     - 3.1.3. Visualization of the anterior border of the upper sacrum.
     - 3.1.4. Reproduction of the vertebral pieces of the upper sacrum.
   - **3.2. Important Image Details**
     - 3.2.1. Linear and reticular details down to 0.5 mm. in width.
   - **3.3. General Assessment**
     - 3.3.1. Image acceptability.

B: Variable definitions of the EU-image criteria and scoring principles for quality

1. LUMBAR SPINE AP/PA Projection (figure 1)

1.1. Image Quality Assessment Criteria *

1.1.1. Visually sharp reproduction of the upper and lower-plate surfaces, represented as lines in the centered beam area.
1.1.2. Visually sharp reproduction of the pedicles.
1.1.3. Reproduction of the intervertebral joints (apophyseal or facet).
1.1.4. Reproduction of the spinous and transverse processes.
1.1.5. Visually sharp reproduction of the cortex and trabecular structures.
1.1.6. Reproduction of the adjacent soft tissues, particularly the psoas shadows.
1.1.7. Reproduction of the sacro-iliac joints.

1.2. Important Image Details**

1.2.1. Image details (3rd lumbar vertebral body) down to 0.3-0.5 mm.

1.3. General Assessment ***

1.3.1. Image acceptability.

1.4. Scoring principles (maximum 11 points)

1.4.1.***Image Quality Assessment Criteria**

Image criteria fulfilled = 1
Image criteria is not fulfilled = 0

1.4.2.**** Important Image Details**

Image details fulfilled = 1
Image details not fulfilled = 0

1.4.3.***** General Image Acceptability**

Fully acceptable = 3
Probably acceptable = 2
Only acceptable under limited clinical conditions = 1, give reasons (<u>see below</u>)
Unacceptable = 0, give reasons (<u>see below</u>)

<u>**Possible reasons for diminished image quality:**</u>

Noise, defined as grainy, random fluctuations in the image which superimpose the image pattern (* = Optimal; - = Suboptimal; 0 = Unacceptable)
Contrast, defined as differences in optical density between object and background (* = Optimal; + = Too high; - = Too low)
Sharpness, defined as the extent of the transition zone for optical density between object and background (* = Optimal; - = Sub-optimal; 0 = Unacceptable)
Collimation, defined as beam limitation (* = Optimal; + = Field size too large; - = Field size too small)
Patient positioning, defined as mal-positioning of the patient in relation to the x-ray field (* = Optimal; - = Sub-optimal; 0 = Unacceptable)

Figure 1:

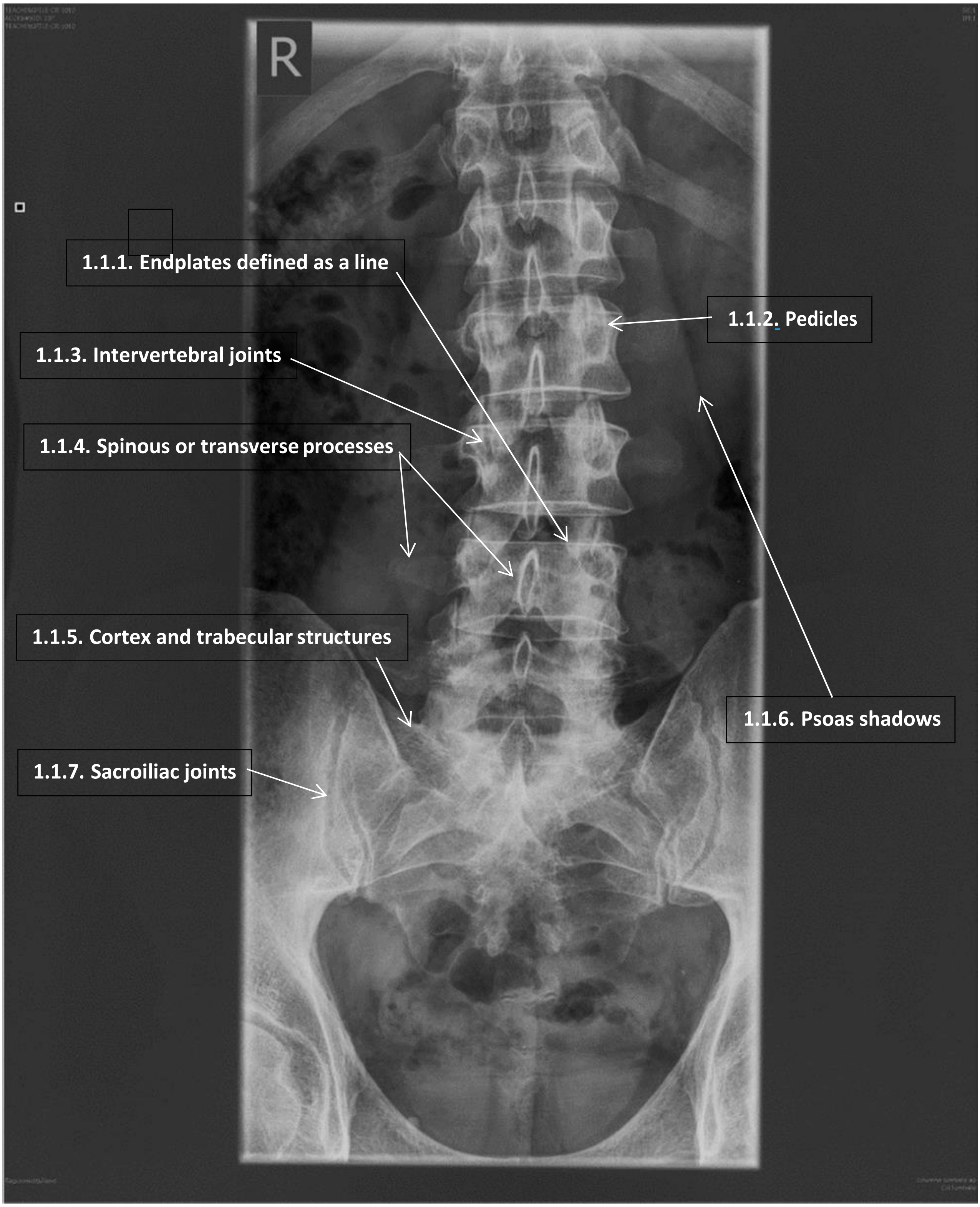
R
1.1.1. Endplates defined as a line
1.1.2. Pedicles
1.1.3. Intervertebral joints
1.1.4. Spinous or transverse processes
1.1.5. Cortex and trabecular structures
1.1.6. Psoas shadows
1.1.7. Sacroiliac joints

2. **LUMBAR SPINE Lateral L1-L4 Projection (figure 2)**

2.1. **Image Quality Assessment Criteria ***

2.1.1. Visually sharp reproduction of the upper and lower-plate surfaces, represented as lines with the resultant visualization of the intervertebral spaces.

2.1.2. Full superimposition of the posterior vertebral edges.

2.1.3. Reproduction of the pedicles and the intervertebral foramina.

2.1.4. Visualization of the spinous processes.

2.1.5. Visually sharp reproduction of the cortex and trabecular structures.

2.2. **Important Image Details****

2.2.1. Visually details down to 0.5 mm. at 3rd lumbar vertebral body, ventral edge.

2.3. **General Assessment *****

2.3.1. Image acceptability.

2.4. **Scoring principles (maximum 9 points)**

2.4.1.***Image Quality Assessment Criteria**

Image criteria fulfilled = 1

Image criteria is not fulfilled = 0

2.4.2.**** Important Image Details**

Image details fulfilled = 1

Image details not fulfilled = 0

2.4.3.***** General Image Acceptability**

Fully acceptable = 3

Probably acceptable = 2

Only acceptable under limited clinical conditions = 1, give reasons (see below)

Unacceptable = 0, give reasons (see below)

Possible reasons for diminished image quality:

Noise, defined as grainy, random fluctuations in the image which superimpose the image pattern (* = Optimal; - = Suboptimal; 0 = Unacceptable)

Contrast, defined as differences in optical density between object and background (* = Optimal; + = Too high; - = Too low)

Sharpness, defined as the extent of the transition zone for optical density between object and background (* = Optimal; - = Sub-optimal; 0 = Unacceptable)

Collimation, defined as beam limitation (* = Optimal; + = Field size too large; - = Field size too small)

Patient positioning, defined as mal-positioning of the patient in relation to the x-ray field (* = Optimal; - = Sub-optimal; 0 = Unacceptable)

Figure 2:

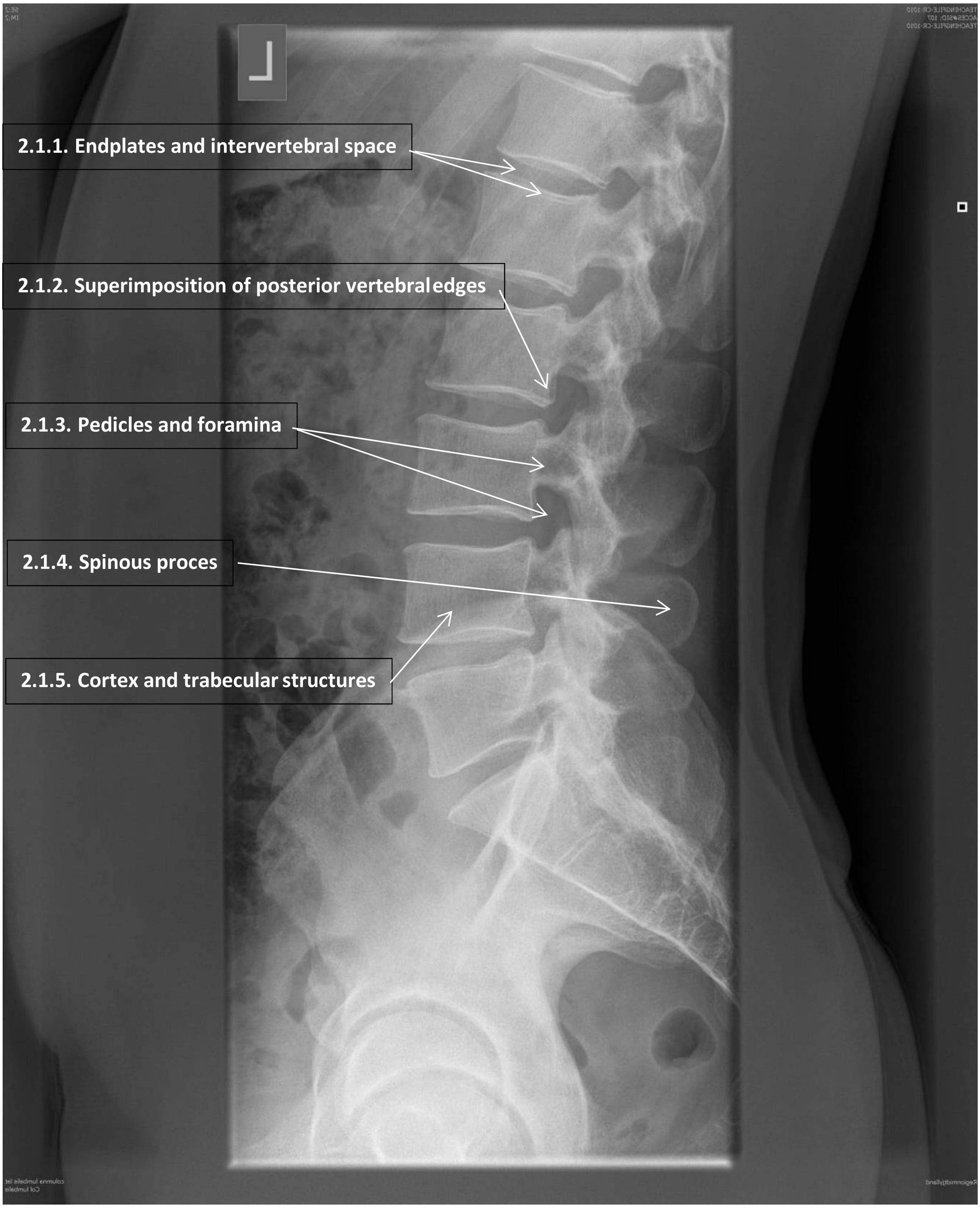
2.1.1. Endplates and intervertebral space
2.1.2. Superimposition of posterior vertebral edges
2.1.3. Pedicles and foramina
2.1.4. Spinous proces
2.1.5. Cortex and trabecular structures

3. **LUMBAR SPINE Lateral L5/S1 Projection (figure 3)**

3.1. **Image Quality Assessment Criteria ***

3.1.1. Reproduction by tangential production of the inferior end plate of L5 and the superior end plate of S1.

3.1.2. Visualization of the spinous process of L5.

3.1.3. Visualization of the anterior border of the upper sacrum.

3.1.4. Reproduction of the vertebral pieces of the upper sacrum.

3.2. **Important Image Details ****

3.2.1. Linear and reticular details down to 0.5 mm. in width.

3.3. **General Assessment *****

3.3.1. Film acceptability.

3.4. **Scoring principles (maximum 8 points)**

3.4.1.***Image Quality Assessment Criteria**

Image criteria fulfilled = 1

Image criteria is not fulfilled = 0

3.4.2.**** Important Image Details**

Image details fulfilled = 1

Image details not fulfilled = 0

3.4.3.***** General Image Acceptability**

Fully acceptable = 3

Probably acceptable = 2

Only acceptable under limited clinical conditions = 1, give reasons (<u>see below</u>)

Unacceptable = 0, give reasons (<u>see below</u>)

<u>**Possible reasons for diminished image quality:**</u>

Noise, defined as grainy, random fluctuations in the image which superimpose the image pattern (* = Optimal; - = Suboptimal; 0 = Unacceptable)

Contrast, defined as differences in optical density between object and background (* = Optimal; + = Too high; - = Too low)

Sharpness, defined as the extent of the transition zone for optical density between object and background (* = Optimal; - = Sub-optimal; 0 = Unacceptable)

Collimation, defined as beam limitation (* = Optimal; + = Field size too large; - = Field size too small)

Patient positioning, defined as mal-positioning of the patient in relation to the x-ray field (* = Optimal; - = Sub-optimal; 0 = Unacceptable)

Figure 3:

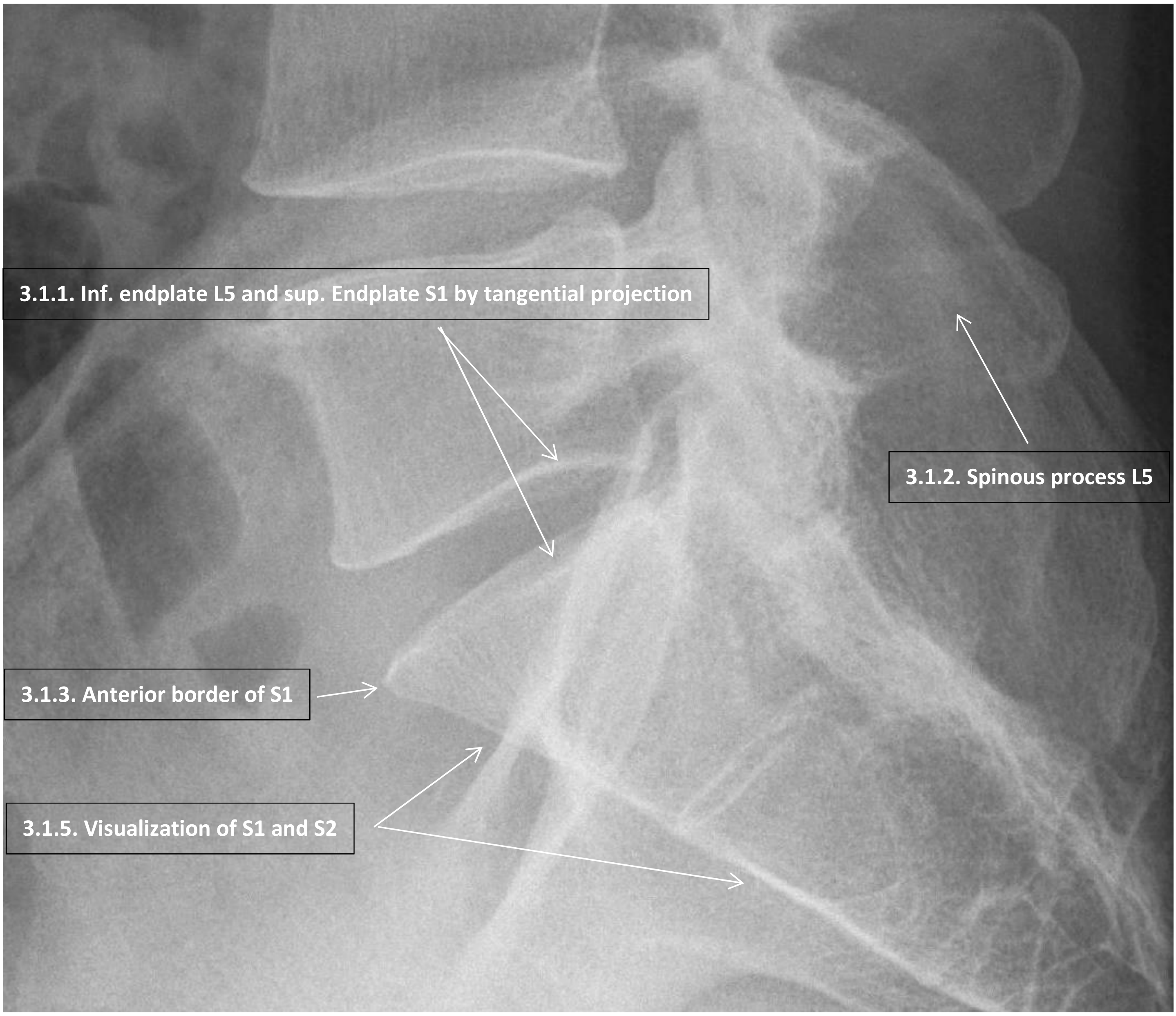

3.1.1. Inf. endplate L5 and sup. Endplate S1 by tangential projection
3.1.2. Spinous process L5
3.1.3. Anterior border of S1
3.1.5. Visualization of S1 and S2
